# Membrane Cholesterol Is Crucial for *Clostridium difficile* Surface Layer Protein Binding and Triggering Inflammasome Activation

**DOI:** 10.3389/fimmu.2020.01675

**Published:** 2020-07-31

**Authors:** Yu Chen, Kai Huang, Liang-Kuei Chen, Hui-Yu Wu, Chih-Yu Hsu, Yau-Sheng Tsai, Wen-Chien Ko, Pei-Jane Tsai

**Affiliations:** ^1^Department of Laboratory Medicine, Mackay Memorial Hospital, New Taipei, Taiwan; ^2^Department of Medical Laboratory Science and Biotechnology, National Cheng Kung University, Tainan, Taiwan; ^3^Department of Pathology, National Cheng Kung University Hospital, Tainan, Taiwan; ^4^Institute of Clinical Medicine, National Cheng Kung University, Tainan, Taiwan; ^5^Department of Internal Medicine, National Cheng Kung University Hospital, Tainan, Taiwan; ^6^Center of Infectious Disease and Signaling Research, National Cheng Kung University, Tainan, Taiwan

**Keywords:** *Clostridium difficile*, membrane cholesterol, lipid rafts, surface layer proteins, inflammasome activation

## Abstract

*Clostridium difficile*, an obligate anaerobic gram-positive bacillus, generates spores and is commonly found colonizing the human gut. Patients with *C. difficile* infection (CDI) often exhibit clinical manifestations of pseudomembranous colitis or antibiotic-associated diarrhea. Surface layer proteins (SLPs) are the most abundant proteins in the *C. difficile* cell wall, suggesting that they might involve in immune recognition. Our previous results demonstrated that *C. difficile* triggers inflammasome activation. Here, we found SLPs as well as *C. difficile* induced inflammasome activation, and in a dose-dependent manner. In addition, the cholesterol-rich microdomains on the cell membrane (also referred to as lipid rafts) are thought to be crucial for bacterial adhesion and signal transduction. We demonstrated that lipid rafts participated in *C. difficile* SLPs binding to the cell membrane. Fluorescence microscopy showed that membrane cholesterol depletion by methyl-β-cyclodextrin (MβCD) reduced the association of SLPs with the cell surface. The coalescence of SLPs in the cholesterol-rich microdomains was confirmed in *C. difficile*-infected cells. Furthermore, the inflammasome activations induced by SLPs or *C. difficile* were abrogated by MβCD. Our results demonstrate that SLPs recruit the lipid rafts, which may be a key step for *C. difficile* colonization and inducing inflammasome activation.

## Introduction

*C. difficile*, an anaerobic gram-positive spore-forming bacillus, is known as one of the most important nosocomial pathogens, and usually causes health care facility-associated infections (1). *C. difficile* is mainly transmitted through the oral–fecal route by spores that are dormant cells, which are highly resistant to the gastric acidic environment (2). Most importantly, *C. difficile* infection (CDI) in the colon is often life-threatening, particularly in the immunocompromised elderly and in patients who have intestinal dysbiosis following antimicrobial drug exposure (3). Moreover, manipulation of toxin secretion and bacterial architecture are crucial for *C. difficile* to colonize the colon, which subsequently induces host inflammation and pathogenesis (4).

Virulent strains of *C. difficile* possess three toxins: toxin A (TcdA), toxin B (TcdB), and a binary toxin (*C. difficile* transferase, CDT), which have been shown to cause bacteria-induced pathogenesis (5). In addition to toxin production, *C. difficile* is found to have surface layers (S-layers) that completely coat the entire vegetative cells and play important roles in bacterial adhesion to enteric cells (6). *C. difficile* S-layers contain two S-layer proteins (SLPs): a conserved high molecular weight (HMW, 42–48 kDa) SLP and a highly variable low molecular weight (LMW, 32–38 kDa) SLP. Both these SLPs are derived from the post-translational cleavage of surface layer protein A (SlpA), which is encoded by a single gene *slpA* (7).

The host innate immune system is the first line of defense against microbial infection and is activated by the engagement of pattern-recognition receptors (PRRs) that are responsible for recognizing specific components expressed by the microbes (8). Previous studies suggested that inflammasome activation is involved in *C. difficile* pathogenesis through several different mechanisms. Ng et al. (9) were the first researchers to demonstrate that inflammasome activation is involved in *C. difficile* infection. Our recent study also demonstrated that the caspase-1-dependent inflammasome plays an important role regulating host defense during *C. difficile* infection (10). Although the molecular mechanism of the *C. difficile* toxin-induction is well-understood, the interactions of this pathogen, either directly or indirectly, with the host innate and adaptive immune system are poorly understood.

Lipid rafts are cholesterol-rich microdomains localized in the cell membrane (11). Several pathogens, including their virulence factors, exploit lipid rafts for entering host cells (12–15). It has been shown that CDT-induced microtubule-based membrane protrusions depend on lipid rafts in enteric cells (16, 17). Moreover, membrane cholesterol is crucial for the delivery of *C. difficile* toxins, TcdA and TcdB, into host cells (18). However, whether SLPs interact with lipid rafts remains to be illustrated. In this study, we explored the involvement of SLPs in triggering inflammasome activation and the association of SLPs with the cell membrane in a cholesterol-dependent manner. We further investigated whether lipid rafts are involved in *C. difficile* induced inflammasome activation.

## Materials and Methods

### Cell Culture

CHO-K1 cells (Chinese hamster ovary cells; ATCC CCL-61) were cultured in F12 medium (Gibco, Grand Island, NY, USA) supplemented with 10% FBS (Biological Industries, Cromwell, CT, USA). The cells were incubated at 37°C in a humid atmosphere containing 5% CO_2_. THP-1 cells (Human acute monocytic leukemia cells) was obtained from Bioresource Collection and Research Center (BCRC), Hsinchu, Taiwan. THP-1 cells were cultured in LPS-free RPMI1640 medium (Gibco) supplemented with 10% FBS (Biological Industries). THP-1 cells were differentiated with 100 nM phorbol 12-myristate 13-acetate (PMA) treatment for 24 h prior to *C. difficile* infection or SLPs exposure.

### Bacterial Culture

*C. difficile* CCUG 37780 (*tcdA*^−^, *tcdB*^−^), a non-toxigenic strain, was cultured on CDC anaerobe 5% sheep blood agar (Becton Dickinson, Cockeysville, MD, USA) in a 37°C incubator for 2 days under the condition anaerobic gas generator (Mitsubishi™ AnaeroPack-Anaero, Japan) as described previously (19). The bacterial colonies were grown in Brain-Heart Infusion (BHI) broth (Becton Dickinson) supplemented with 5 mg/ml yeast extract and 0.1% L-cysteine (Amresco, Solon, OH, USA) at 37°C for 2 days. The bacteria were washed with 1 × PBS prior to macrophage infection.

### Preparation of Surface Layer Proteins (SLPs)

The extraction method was modified from a previous study (20). Briefly, overnight culture of *C. difficile* was collected, and the SLPs was extracted by 0.2 M glycine, pH 2.2. After removing the bacterial components, the supernatant was neutralized with 2 M Tris-HCI. To increase the purity of SLPs, the neutralized supernatant was filtered by 50 kDa molecular weight-cutoff centrifugation-based filters (Millipore). Then the filtrate was concentrated by 30 kDa centrifugation-based filters (Millipore).

### SDS-PAGE and Western Blot Assays

Purified SLPs were boiled in SDS-PAGE sample buffer for 10 min and subjected to 10% SDS-PAGE. The gel was stained with Coomassie Brilliant Blue R-250 (Amresco) for visualization of SLPs. In addition, the gel was transferred onto polyvinylidene difluoride membranes (PVDF, Millipore, Billerica, MA, USA). The membranes were blocked with TBST containing 5% skim milk for 1 h and then incubated with anti-SLPs antibody followed by incubation with HRP-conjugated secondary antibodies (Millipore) for 1 h. The proteins of interest were detected using ECL western blotting detection reagents (GE Healthcare, Chicago, IL, USA), and were visualized using Azure c400 system and AzureSpot Analysis Software (Azure Biosystems; Dublin, CA, USA) according to the manufacturer's instructions.

For monitoring caspase-1 and IL-1β maturation, total proteins were separated by SDS-PAGE, transferred to PVDF membranes, and probed with antibodies against IL-1β (R&D system, Minneapolis, MN, USA), precursor and p10 subunit of caspase-1 (Abcam, Cambridge, United Kingdom) and β-actin (Sigma-Aldrich, St. Louis, Missouri, USA). The expression of low-molecular-weight (LMW) surface layer proteins in the cell culture supernatant was also detected using rabbit anti-LMW SLP BAA 1805 serum (customized by Abnova, Taipei, Taiwan).

### Flow Cytometric Analysis

To analyze the binding of SLPs to lipid rafts, CHO-K1 cells treated with *C. difficile* SLPs were analyzed by flow cytometry. CHO-K1 cells (7 × 10^5^) were pretreated with 10 mM methyl-β-cyclodextrin (MβCD, Sigma-Aldrich Saint Louis, MO, USA), a cholesterol depletion agent commonly used to extract cholesterol from lipid rafts, for 1 h, and then treated with SLPs (20 μg/ml) at 4°C for an additional 2 h. The green fluorescent intensity of SLPs probed with fluorescein isothiocyanate (FITC)-conjugated goat anti-rabbit IgG was analyzed by flow cytometry (FACSCalibur Cell Analyzer, Becton Dickinson).

### Immunofluorescence Microscopy

To visualize the localization of SLPs on cell membrane, CHO-K1 cells (1 × 10^6^) were seeded on coverslips in a 6-cm dish and incubated for 10 h. Cells were pretreated with 10 mM MβCD and then exposed to SLPs (20 μg/ml) at 11°C for 1 h to maintain the fluidity of the cell membrane and to prevent the internalization of cells. Cells were infected with *C. difficile* at a MOI of 20 and incubated at 37°C for 6 h. The treated cells were then washed with 1 × PBS and fixed with 4% paraformaldehyde (Sigma-Aldrich). The cells were probed with anti-caveolin-1-conjugated FITC antibody (Santa Cruz Biotechnology, Santa Cruz, CA), which is a marker for staining membrane rafts. The cells were then incubated with anti-SLP antibody, followed by Alexa Fluor red 555-conjugated goat anti-rabbit IgG (Invitrogen, Carlsbad, CA). Nuclei were counterstained with Hoechst 33,342. The samples were observed under a confocal laser-scanning microscope (LSM780, ZEISS, Germany). The quantification of fluorescence intensity for SLPs and caveolin-1 was analyzed using ZEN software (Carl Zeiss, Göttingen, Germany).

### Statistical Analysis

Experimental results are expressed as mean ± SEM. Student's *t*-test was used to determine the statistical significance of the differences between two groups. Differences were considered significant when *P* < 0.05. Statistical analysis was performed using Prism6 (GraphPad Software, La Jolla, CA, USA).

## Results

### *C. difficile* SLPs Induce Inflammasome Activation

**Figure 1:**
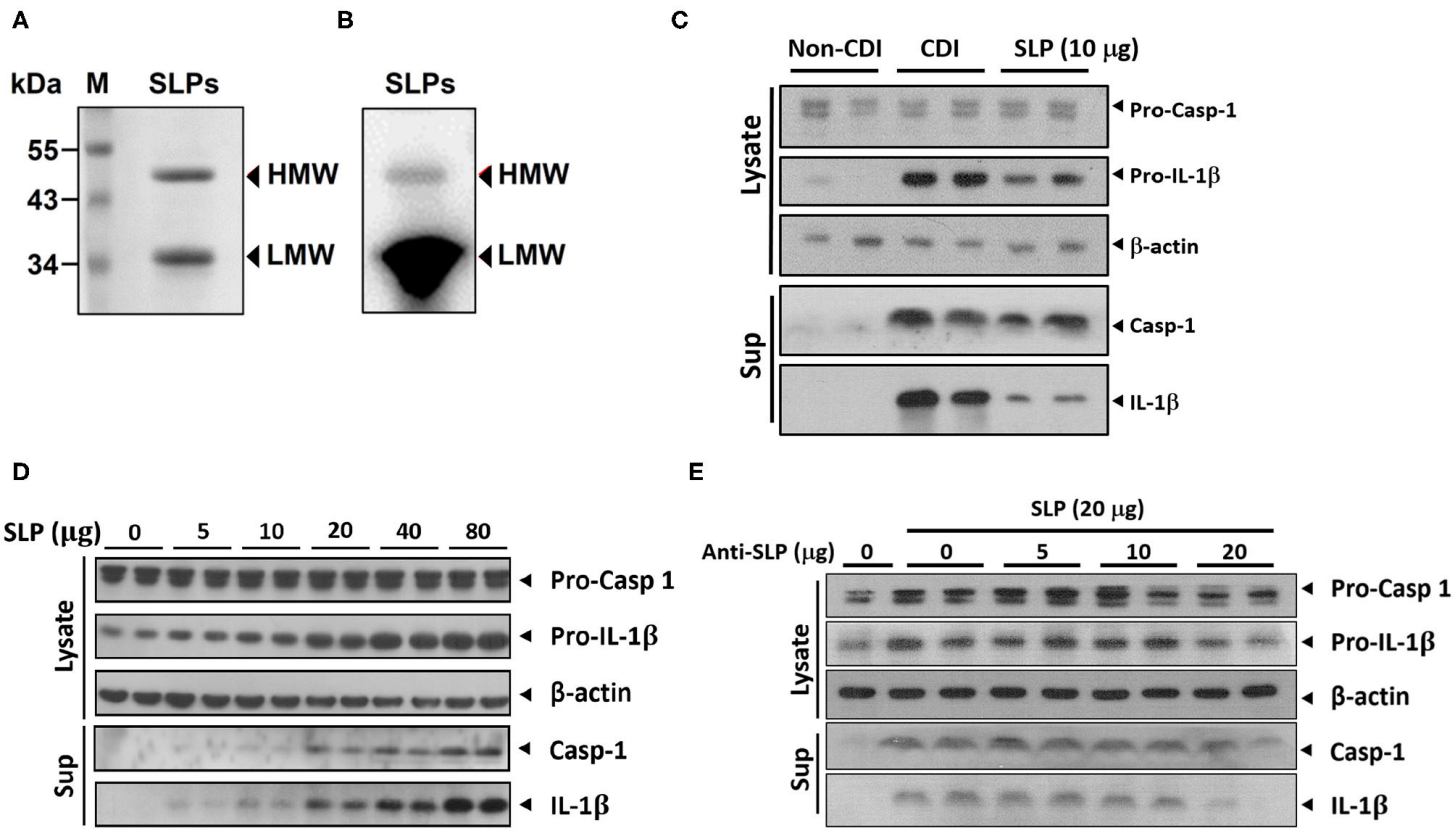
SLPs of *C. difficile* induced inflammasome activation. **(A)** SLPs were isolated from non-toxigenic *C. difficile* (*tcdA*^−^, *tcdB*^−^), followed by **(A)** SDS-PAGE and **(B)** western blot assays. Purified SLPs possess high molecular weight (HMW) and low molecular weight (LMW) components. M, protein markers with molecular weights in kDa. The production of matured caspase-1 and IL-1β were increased by SLP treatment as well as *C. difficile* infection **(C)**, and in a dose-dependent manner **(D)**. **(E)** Matured caspase-1 and IL-1β were blockaded by anti-SLP Abs.

Previous studies suggested that SLPs of *C. difficile* could induce production of proinflammatory cytokines including IL-1β (21). Therefore, we tried to examine whether SLPs induce inflammasome activation. We purified SLPs, including low and high molecular weight SLPs from the non-toxigenic *C. difficile* strain CCUG 37780. The purified SLPs were analyzed using 10% SDS-PAGE and western blot assays. As shown in Figures 1A,B, *C. difficile* SLPs contained two components: HMW and LMW with molecular weights of 45 and 34 kDa, respectively. After treating THP-1 cells with purified SLPs or *C. difficile* infection (CDI), the production of matured caspase-1 and IL-1β were increased when compared to non-CDI control group (Figure 1C). Noticeably, SLPs-induced caspase-1 and IL-1β production were in dose-dependent manners (Figure 1D). Moreover, blockage of SLPs with SLP antibody dose-dependently decreased the production of caspase-1 and IL-1β (Figure 1E). These results showed that SLPs can induce inflammasome activation.

### *C. difficile* SLPs Bind to the Cell Membrane

**Figure 2:**
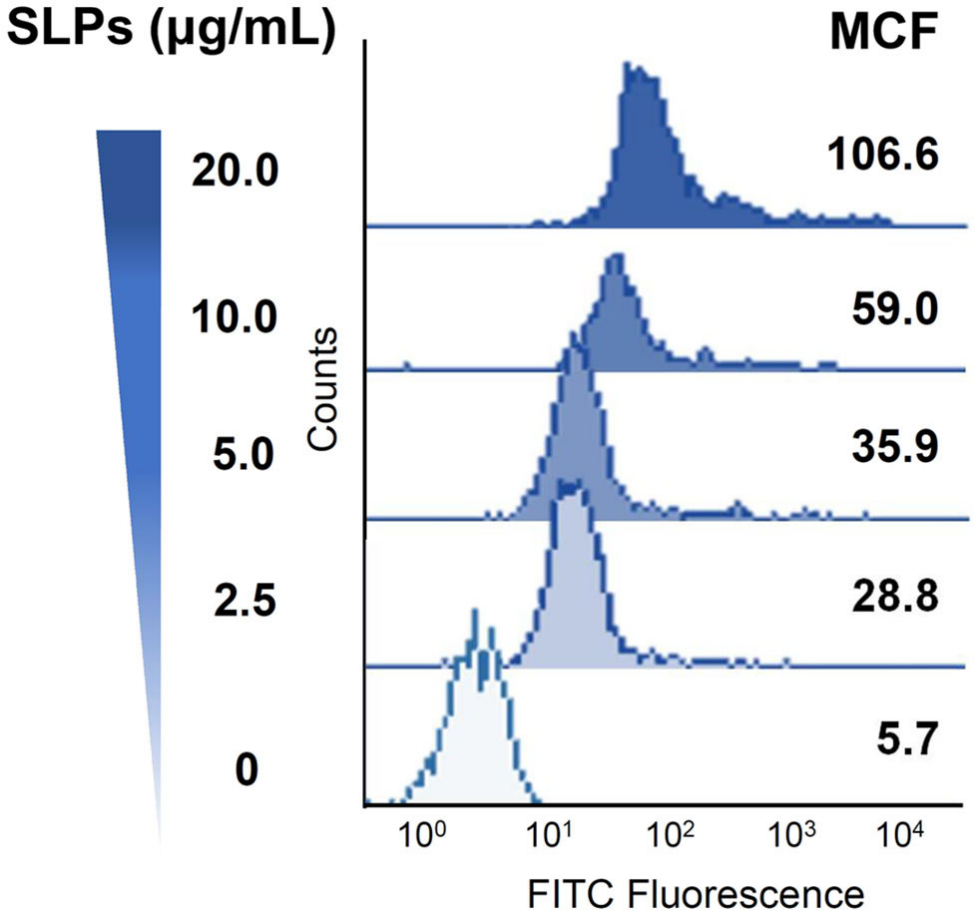
Binding of *C. difficile* SLPs to cells. CHO-K1 cells were exposed to various concentrations of SLPs (0–20 μg/ml) and incubated at 4°C for 2 h. The cells were probed with the anti-SLP antibody at 4°C for 2 h, followed by incubation with FITC-conjugated goat anti-rabbit IgG. The binding activity of SLPs was assessed using flow cytometry to detect FITC fluorescence intensity. MCF, mean channel fluorescence.

We then investigated whether *C. difficile* SLPs interact with the cell membrane; CHO-K1 cells were used as assay platforms. Cells were treated with various concentrations of SLPs at 4°C for 2 h. The treated cells were then analyzed using flow cytometry. Figure 2 showed that with the elevation of SLP concentrations (0–20 μg/ml), the binding of SLPs on the cell membrane gradually increased.

**Figure 3:**
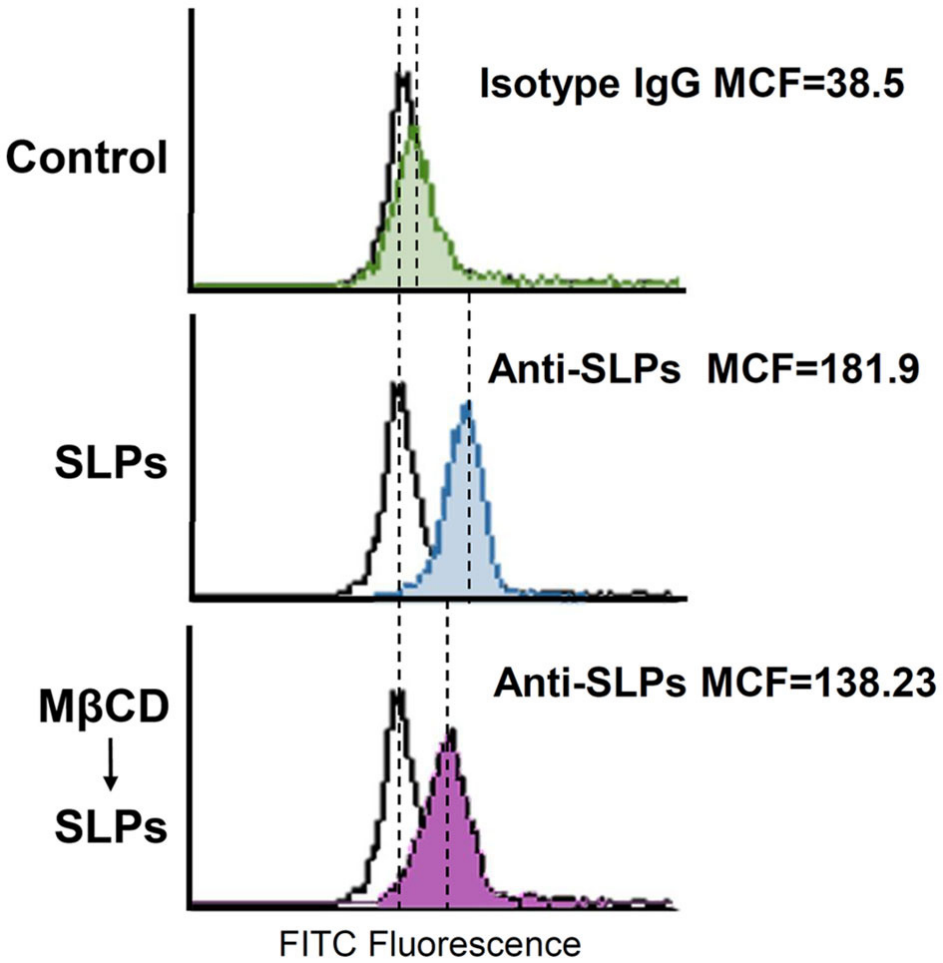
Sufficient cholesterol is essential for SLP binding to cells. CHO-K1 cells were untreated or pretreated with 10 mM MβCD at 37°C for 1 h, followed by exposure to SLPs (20 μg/ml). After treatment with the anti-SLP antibody at 4°C for 2 h, the cells were stained with FITC-conjugated goat anti-rabbit IgG. The binding activity of SLPs was assessed using flow cytometry to detect FITC fluorescence intensity. MCF, mean channel fluorescence.

We next explored whether cholesterol depletion affects the binding of SLPs to cells. CHO-K1 cells were pretreated with MβCD at 37°C for 1 h to deplete cholesterol from cells. As shown in Figure 3, the mean channel fluorescence (MCF) for SLP binding to cells was reduced in cells pretreated with MβCD as compared to that in the untreated control cells. These results indicate that SLPs bind to the membrane where the lipid rafts are localized.

### Depletion of Cellular Cholesterol Reduces SLP Binding to the Cell Membrane

**Figure 4:**
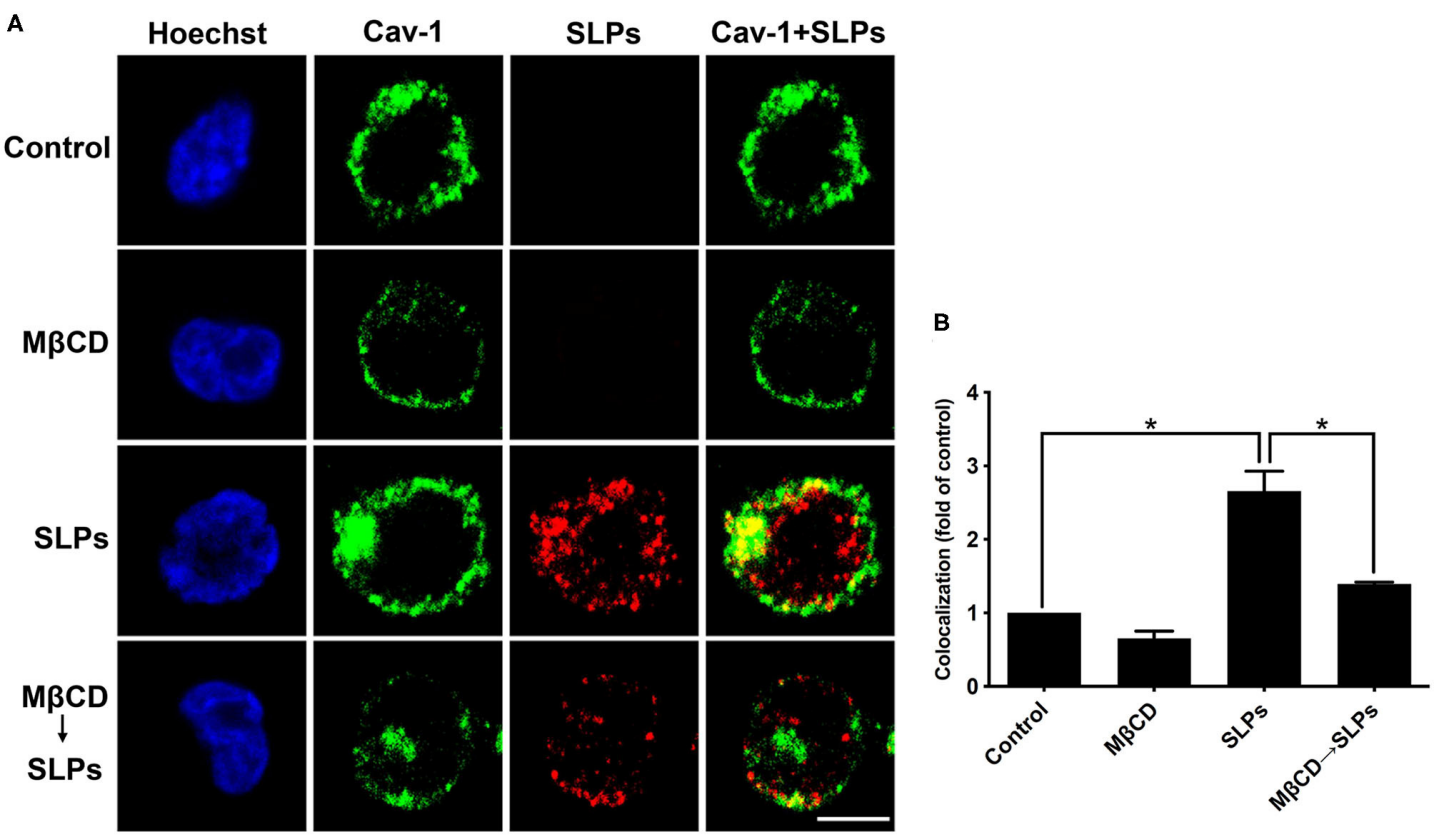
Interaction of SLPs with lipid rafts. **(A)** CHO-K1 cells were untreated or pretreated with 10 mM MβCD for 1 h at 37°C prior to incubation with SLPs (20 μg/mL) at 11°C for 1 h. Cells were fixed with 4% paraformaldehyde and stained with anti-caveolin-1-conjugated FITC antibody (green). Cells were then incubated with the anti-SLP antibody, followed by Alexa Fluor red 555-conjugated goat anti-rabbit IgG (red). Cells were finally probed with Hoechst 33,342 for nuclei staining (blue). The stained cells were then analyzed using confocal microscopy. The colocalization of SLPs with caveolin-1 appears yellow in the overlay. Scale bar, 10 μm. Cav-1, caveolin-1. **(B)** The fluorescence intensity of caveolin-1 and SLP was analyzed using the ZEN software (Carl Zeiss). Colocalized punctate of caveolin-1 with SLPs was quantified using merged pixels and normalized to those in the control group. Statistical analysis was calculated using Student's *t*-test. *P < 0.05 was considered statistically significant.

We used confocal microscopy to visualize whether SLP binding to cells is dependent on the lipid rafts. CHO-K1 cells were either untreated or pretreated with 10 mM MβCD for 1 h at 37°C, prior to exposure to 20 μg/ml SLPs at 11°C for 1 h. Cells were probed with the anti-caveolin-1 antibody to identify the membrane raft microdomains. As shown in Figure 4, the control cells untreated with SLPs did not show red fluorescence signal; however, caveolin-1 (green) was observed around the membrane (first row). When cells were pretreated with 10 mM MβCD, cell membrane caveolin-1 was reduced (second row). Cells incubated with SLPs showed considerable colocalization with the membrane raft marker caveolin-1 (third row, merged in yellow). After pretreating cells with MβCD to deplete cholesterol, followed by incubation with SLPs, the overlay of the yellow fluorescent signal was weaker (fourth row) than that in cells untreated with MβCD. These results indicate that SLPs isolated from *C. difficile* possess binding activity to lipid rafts on the cell surface.

### Interaction of *Clostridium difficile* SLPs With Membrane Rafts

**Figure 5:**
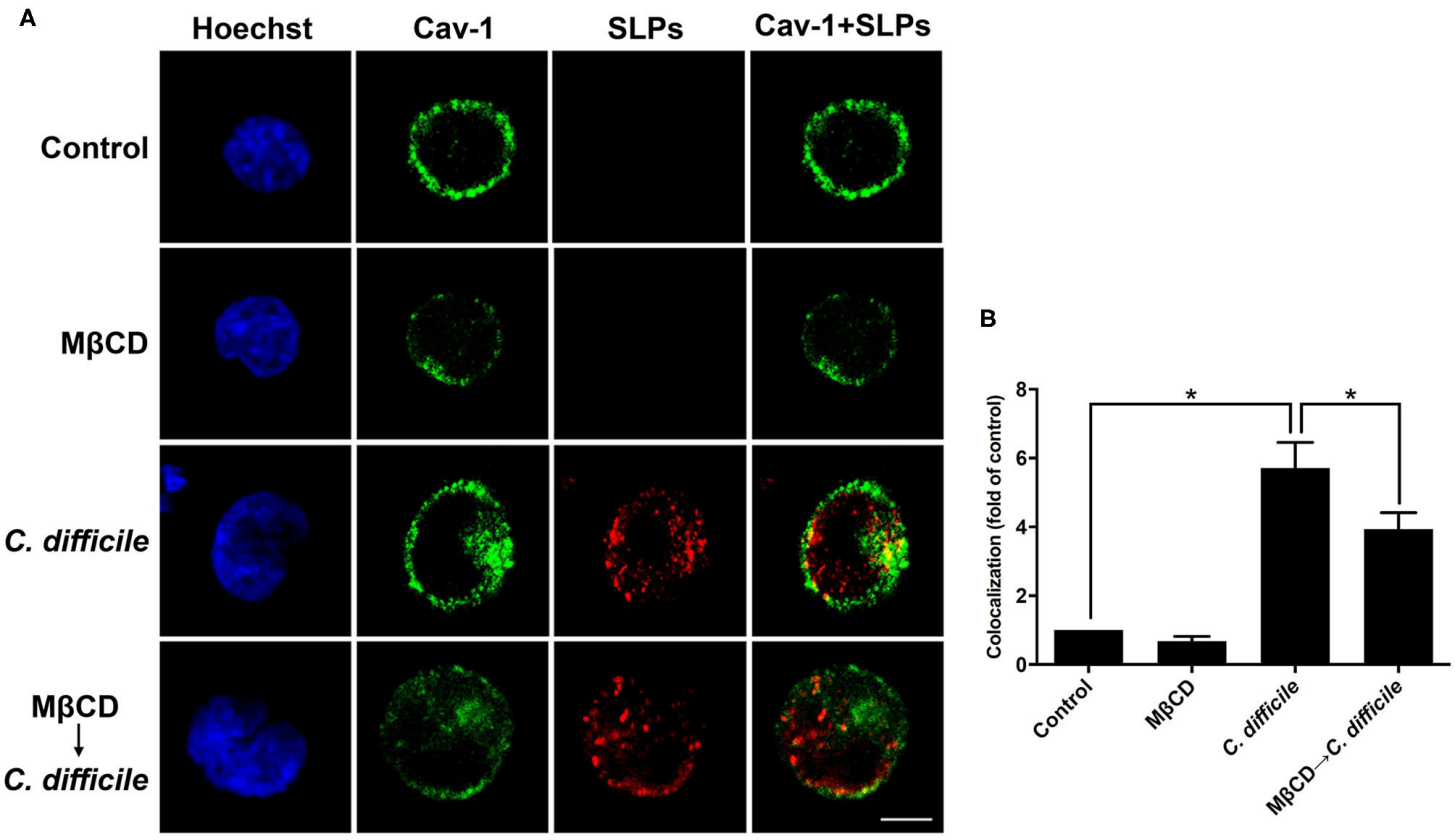
Localization of SLPs in lipid rafts of *C. difficile-*infected cells. **(A)** CHO-K1 cells were untreated or pretreated with 10 mM MβCD for 1 h prior to infection with non-toxigenic strain *C. difficile* (CCUG37780) at 37°C for 6 h. Cells were fixed and then probed with anti-caveolin-1 conjugated FITC antibody (green) and anti-SLPs antibody, followed by staining with Alexa Fluor red 555-conjugated goat anti-rabbit IgG (red), and Hoechst 33,342 for nuclei (blue). The samples were then analyzed by confocal microscopy. The colocalization of SLPs with caveolin-1 appears yellow in the overlay. Scale bar, 10 μm. **(B)** The fluorescence intensity of caveolin-1 and SLPs was analyzed using ZEN software (Carl Zeiss). Colocalized punctate of caveolin-1 and SLPs was quantified using merged pixels and normalized to those in the control group. Statistical analysis was calculated using Student's *t*-test. *P < 0.05 was considered statistically significant.

We then used cells infected with *C. difficile* to verify the interactions between bacterial SLPs and lipid rafts. In the absence of *C. difficile* infection, there was no SLP signal (Figure 5). *C. difficile* infected cells showed that SLPs were abundantly distributed around the cytoplasm as well as the membrane, as indicated by the colocalized caveolin-1. In contrast, pretreatment of cells with 10 mM MβCD reduced the colocalization signals of SLPs and caveolin-1. These results demonstrate that upon infection by *C. difficile*, SLPs bind to the membrane that has cholesterol-rich microdomains.

### Depletion of Cellular Cholesterol Diminishes the Inflammasome Activation Induced by SLPs and *C. difficile*

**Figure 6:**
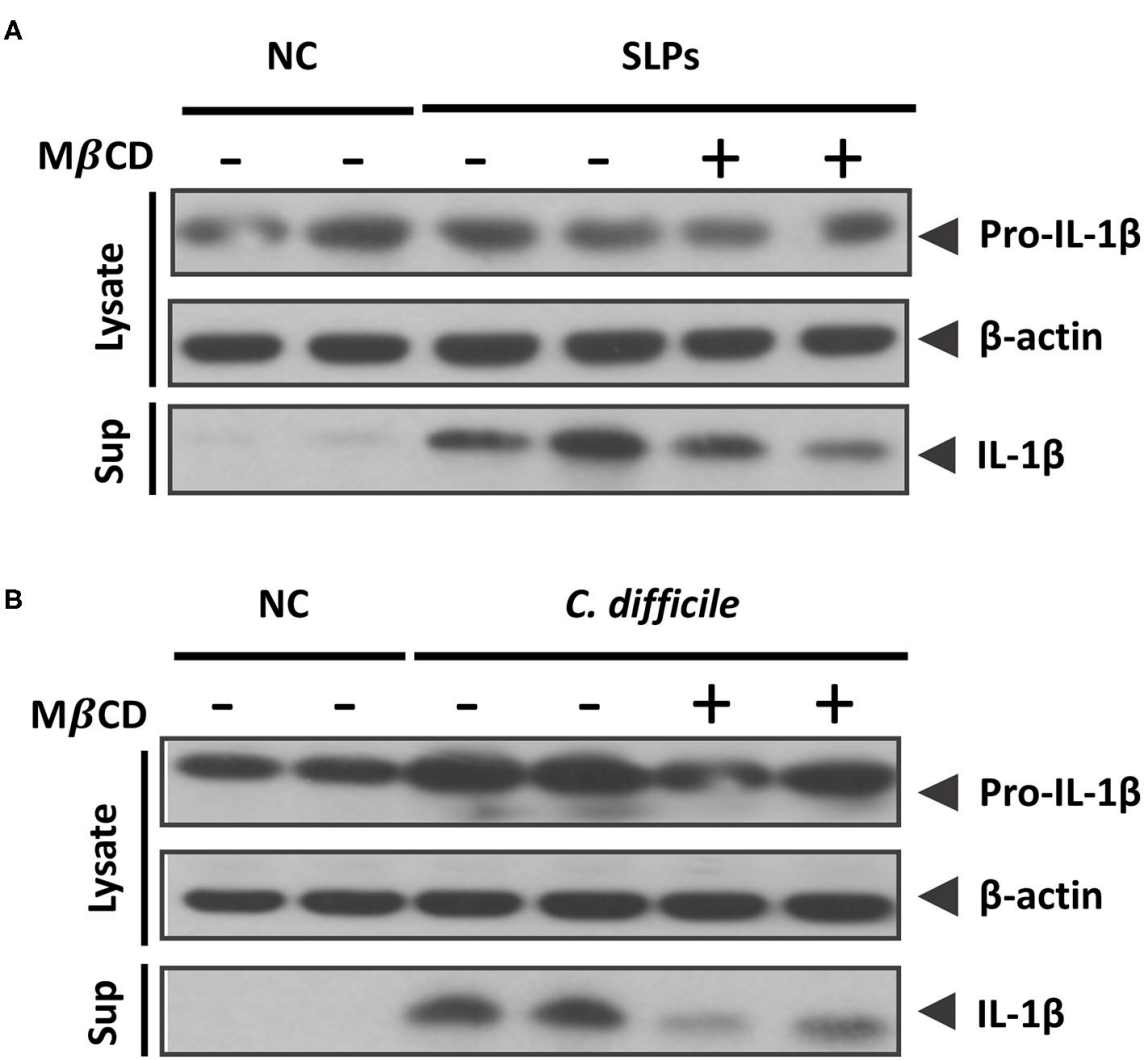
Sufficient cholesterol is essential for *C. difficile* and SLPs-induced inflammasome activation. Western blot analysis of **(A)** IL-1β maturation either in cell lysate or supernatant after SLPs treatment, and **(B)** *C. difficile* infection.

To connect the relationship between inflammasome activation induced by SLPs or *C. difficile* with lipid rafts, MβCD pretreated cells were examined for the inflammasome activation. In the SLPs treated cells showed the abundant proinflammatory cytokine IL-1β upon compared to the negative control (NC), and the activation of the inflammasome was reduced while the cells were depleted cholesterol by MβCD (Figure 6A). In addition, we further investigated the inflammasome activation triggered by *C. difficile* infection. The results showed that the *C. difficile* infection can trigger IL-1β maturation and this activation was reduced by the pretreatment with MβCD (Figure 6B). Both results demonstrate that the SLPs as well as *C. difficile* induced the inflammasome activation mediated through the lipid rafts.

## Discussion

SLPs provide structural integrity to the bacterial cells and participate in the adhesion to host cells (22–24). Although SLPs have been recognized as virulence factors for bacteria-induced pathogenesis, the receptors for SLP binding to cells need to be explored. In this study, we found that cellular cholesterol plays a pivotal role in *C. difficile* SLP binding and triggering inflammasome activation. Investigating the interactions between bacterial virulence factors and cell receptors is particularly crucial because these key molecules have been proposed as possible targets for treating bacterial infectious diseases.

Purified SLPs from *C. difficile* can be recognized by Toll-like receptor 4 (TLR4), which activates inflammatory responses and contributes to CDI pathogenesis (25). Moreover, SLPs of *C. difficile* are capable of inducing proinflammatory cytokines, including IL-1β, IL-12, IL-23, and TNF-α (21, 26). Our recent study further demonstrated that SLPs were released from damaged cells via caspase-1-mediated regulation (10). These findings indicate that SLPs possess the ability to orchestrate the immune response during CDI pathogenesis. Furthermore, we also discovered that inflammasome activation was induced by SLPs during *C. difficile* infection, suggesting that the SLPs play an important role in regulating host defense against *C. difficile* infection.

Lipid rafts on the cell membrane are enriched in cholesterol and sphingolipids, and play crucial roles in various cellular processes, including membrane trafficking, signal transduction, cytoskeletal rearrangement, and pathogen entry (27–29). Most importantly, lipid rafts also function as internalized portals for the entry of bacterial toxins, which contribute to severe infectious diseases including shigellosis and anthrax (12, 30). The study on *C. difficile* toxin has demonstrated that CDT-induced microtubule-based membrane protrusions were dependent on cholesterol-rich microdomains present in the human colon cells (16). Moreover, the active subunit of CDT, called CDTb, that binds to the lipolysis-stimulated lipoprotein receptor (LSR) was found to coalesce into lipid rafts (17). In this study, we further demonstrated that *C. difficile* SLPs interact with the cell membrane rich in lipid rafts. Together, these findings indicate that both CDT and SLPs interact with raft-microdomains, subsequently inducing the *C. difficile* infection process and pathogenic response in the intestinal epithelial cells.

Colonization is an essential process that interferes with the gut microbiota in *C. difficile*-induced pathogenesis (31). Many bacterial surface proteins contribute to the colonization of the host intestinal epithelium and subsequent multiplication in the gut surface and lumen (32). This study employed Chinese hamster ovary-K1 (CHO-K1) cells to investigate the association of *C. difficile* SLPs with the cell membrane. Our results showed that SLPs are capable of binding to the cell surface, in which activity was decreased when cellular cholesterol was depleted using MβCD. Many studies have used CHO-K1 cells as an assay model to analyze membrane-raft functions (33–35). Therefore, it appears that CHO-K1 is a suitable model for exploring the relationship between *C. difficile* SLPs and lipid rafts. Interestingly, it has been reported that CHO-K1 cells lack surface Toll-like receptors (36, 37). We speculate that in addition to TLR4, other molecules are involved in the mechanism of *C. difficile* SLP attachment to host cells.

Although *C. difficile* toxins are considered crucial for disease development, previous studies have reported that non-toxigenic *C. difficile* is also present in the stool samples of hospitalized patients with persistent diarrhea (38). Our results indicate that regardless of purified SLPs or direct infection of *C. difficile* to cells, similar trends were observed, which demonstrated that SLPs and lipid rafts are important for the *C. difficile*-induced inflammasome activation. This evidence further indicates that besides toxins, SLPs are also involved in *C. difficile*-associated diseases. However, the in-depth study of how *C. difficile* SLPs interact with lipid rafts has been hampered by the inability to establish a *slpA*-deficient mutant strain, which is the limitation of this study as well. Therefore, to overcome this limitation, a stable *C. difficile* SLP-knockout strain is needed for further study. On the other hand, statins, a class of lipid-lowering medications, have pleiotropic effects beyond cholesterol lowering by immune modulation (39). The association of statins with CDI is unclear as clinical studies have reported conflicting findings (40, 41). Modifying the lipid rafts during CDI may provide potentially important novel alternative therapeutic targets to treat and prevent *C. difficile* infection.

## Conclusion

This study demonstrates that membrane cholesterol plays important roles in *C. difficile* SLPs binding and triggering inflammasome activation. Disruption of lipid rafts reduces SLPs binding to cells and mitigates *C. difficile*-induced inflammasome activation. The membrane receptors that contribute to SLPs interaction with cholesterol-rich microdomains will be the subject of research in further studies. This novel discovery contributes to understanding the essential factors for *C. difficile* infection and enable the development of novel therapeutic strategies to prevent CDI.

## Data Availability Statement

All datasets presented in this study are included in the article.

## Author Contributions

YC, KH, L-KC, and H-YW researched data, contributed to the discussion, and wrote the manuscript. Y-ST, C-YH, and W-CK contributed to the discussion, and reviewed and edited the manuscript. P-JT contributed to the original concept and discussion, and wrote, reviewed, and edited the manuscript. All authors contributed to the article and approved the submitted version.

## Conflict of Interest

The authors declare that the research was conducted in the absence of any commercial or financial relationships that could be construed as a potential conflict of interest.
